# Challenges and opportunities in characterizing cognitive aging across species

**DOI:** 10.3389/fnagi.2012.00033

**Published:** 2012-11-26

**Authors:** Thomas C. Foster

**Affiliations:** Department of Neuroscience, University of FloridaGainesville, FL, USA

With the prolonged lifespan brought about by medical advances, attention is increasingly focused on problems that arise from such longevity, including diseases that tend to occur in elderly populations. Despite progress on health related issues, little progress has been made with respect to the treatment of cognitive decline that can accompany the aging process. In humans, age-related impairments can become apparent in middle-age and progress with advancing age. However, not all cognitive and behavioral processes are equally vulnerable to senescence. An important aspect of defining normal cognitive aging is to emphasize the distinction between age-related deficits not specifically associated with disease, and those that do result from neurological diseases (such as Alzheimer's disease). It is important to recognize that within the population of disease free elderly; there is considerable variability in cognition. This fact suggests that understanding the biological or physiological aging of specific brain systems, rather than using chronological age alone as the defining variable, is key to understanding individual differences and successful trajectories in cognitive aging.

This Special Issue, focused on “Challenges and opportunities in characterizing cognitive aging across species” is the result of the McKnight Brain Research Foundation working group meetings dedicated to examining a library of age-sensitive tasks that can be used to translate findings across different levels of analysis and across different species. The aim of this work is to allow researchers to draw more accurate conclusions about human cognitive aging from work in other model species. Additionally, this work highlights gaps in linking human and animal laboratory models, and recommends approaches for filling those gaps.

The introductory article by Roberson et al. (2012), discusses the translational aspect of behavioral techniques used in measuring cognitive function in animals to humans. The authors present the criteria employed by the working group for selection of models and tests for consideration, as well as highlighting the challenges and opportunities in characterizing cognitive aging across species. The next four manuscripts examine specific cognitive processes, linked to defined neural systems, and identify tests that are useful in both humans and animal experimental models. Engle and Barnes (2012) provide an overview of single-cue delay and trace eyeblink conditioning paradigms to assess associative learning and memory during aging, discussing potential confounds, solutions, and optimization of these protocols. Eyeblink conditioning is an especially powerful model of associative learning because of the relative ease in employing this technique across species, including humans. Burke et al. (2012) describe converging evidence from rats, monkeys, and humans to argue that senescence of the perirhinal cortex mediates a decline in recognition memory and complex perceptual processing. The authors suggest that further investigation of perirhinal function in aging represents an important avenue for future research toward potential new therapies to alleviate age-related cognitive decline. Episodic memory is particularly vulnerable to decline in aging humans and Foster et al. (2012) examine cross-species considerations for deficits in hippocampal-dependent spatial and contextual memory. They describe variations in training procedures used to track a progressive decline in rapid, flexible spatial learning and memory to impairment in the incremental acquisition of spatial reference memories. Their discussion on virtual environment testing in humans provides a novel contribution to the field. Bizon et al. (2012), provide an extensive review of memory and executive function processes in aging and emphasize impairments in processes that parallel human aging. Evidence is provided to indicate that alterations in executive functions can mediate widespread deficits on a diverse array of neurocognitive processes and they discuss how these aspects can be quantified in rodents.

The final two articles explore research directed at cognitive aging in humans. The first article by Geldmacher et al. (2012), provides a guide for researchers interested in conducting research using healthy aging populations. Barriers to the study of cognitive aging, including demographics and medical status, including dementia, are discussed. Alexander et al. (2012), provide a comprehensive review of approaches that have been applied in human research studies to evaluate the effects of aging on cognition and discuss examples from experimental and clinical approaches for examining age-related changes in executive function, memory, processing speed, language, and visuospatial function.

Together these seven articles emphasize essential concerns for understanding and interpreting results from human studies of cognitive aging and address the aspects of aging model systems that are important in relating non-human species to human cognitive senescence. The ideas advanced in these articles provide a basis for tests that are valid for cognitive domains across species, permitting progress in research on basic mechanisms and potential treatments of cognitive decline.
